# Serum Phospholipid Profile Changes in Gaucher Disease and Parkinson’s Disease

**DOI:** 10.3390/ijms231810387

**Published:** 2022-09-08

**Authors:** Laura López de Frutos, Francisco Almeida, Jessica Murillo-Saich, Vasco A. Conceição, Monica Guma, Oswald Queheberger, Pilar Giraldo, Gabriel Miltenberger-Miltenyi

**Affiliations:** 1 Fundación para el Estudio y la Terapéutica de la Enfermedad de Gaucher y Otras Lisosomales (FEETEG), 50006 Zaragoza, Spain; 2 GIIS-012, Instituto de Investigación Sanitaria Aragón (IIS Aragón), Unidad de Investigación Traslacional, Hospital Universitario Miguel Servet, 50009 Zaragoza, Spain; 3 Instituto de Medicina Molecular João Lobo Antunes, Faculdade de Medicina, Universidade de Lisboa, 1649004 Lisbon, Portugal; 4 Department of Medicine, University of California San Diego, La Jolla, CA 92093, USA; 5 VA Medical Center, San Diego, CA 92093, USA; 6 Department of Medicine, Autonomous University of Barcelona, 08193 Bellaterra, Spain; 7 Department of Pharmacology, School of Medicine, University of California San Diego, La Jolla, CA 92093, USA; 8 Laboratório de Genética, Faculdade de Medicina, Universidade de Lisboa, 1649004 Lisbon, Portugal; 9 Department of Neurology, Ludwig-Maximilians-Universität München, 80539 Munich, Germany; 10 Genetics Department, Reference Center on Lysosomal Storage Disorders, Hospital Senhora da Oliveira, 4835-044 Guimarães, Portugal

**Keywords:** Parkinson’s disease, Gaucher disease, plasma phospholipids, GBA1, miglustat, dopamine agonist

## Abstract

Alterations in the levels of serum sphingolipids and phospholipids have been reported in Gaucher disease and in Parkinson’s disease, suggesting a potential role of these lipids as biomarkers. This project’s objective is to detect novel associations and novel candidate biomarkers in the largest Spanish Gaucher and Parkinson diseases of the Iberian Peninsula. For that, 278 participants were included: 100 sporadic Parkinson’s patients, 70 Gaucher patients, 15 *GBA1*-mutation-carrier Parkinson’s patients and 93 controls. A serum lipidomics array including 10 phospholipid groups, 368 species, was performed using high-performance liquid chromatography–mass spectrometry. Lipid levels were compared between groups via multiple-regression analyses controlling for clinical and demographic parameters. Additionally, lipid levels were compared within the Gaucher and Parkinson’s groups controlling for medication and/or disease severity. Results were controlled for robustness by filtering of non-detectable lipid values. There was an increase in the levels of phosphatidylcholine, with a simultaneous decrease in lyso-phosphatidylcholine, in the Gaucher, Parkinson’s and *GBA1*-mutation-carrier Parkinson’s patients vs. controls. Phosphatidylethanolamine, lyso- and plasmalogen-phosphatidylethanolamine were also increased in Gaucher and Parkinson’s. Gaucher patients also showed an increase in lyso-phosphatidylserine and phosphatidylglycerol. While in the Gaucher and Parkinson’s groups, velaglucerase alpha and dopamine agonists, respectively, showed positive associations with the lipid changes, miglustat treatment in Gaucher patients normalized the altered phosphatidylcholine/lyso-phosphatidylcholine ratio. In conclusion, Gaucher and Parkinson’s patients showed changes in various serum phospholipid levels when compared with healthy controls, further supporting the role of such lipids in disease development and, possibly, as putative biomarkers. This hypothesis was reinforced by the normalizing effect of miglustat, and by controlling for data robustness, even though the limited number of participants, especially in the sub-distribution by treatment groups in GD requires validation in a larger number of patients.

## 1. Introduction

In the recent years, various studies reported on sphingolipids and—with less extent—phospholipids as potential diagnostic and prognostic biomarkers for the lysosomal storage diseases (LSDs) Gaucher disease (GD) [1,2], Fabry disease [3], and Acid Sphingomyelinase Deficiency (Niemann-Pick disease type A and B) [4]. Less is known about serum phospholipids even though its role in the pathomechanism of GD is plausible [5,6].

GD is the most common LSD, caused by bi-allelic mutations in the *GBA1* gene (MIM*606463) that encodes for the lysosomal glucocerebrosidase (GCase, EC:3.2.1.45). GD is classified according to the neurological involvement as (i) type 1 (GD1; adult form; MIM#230800) being the most common form of GD with no neurological damage; (ii) type 2 (GD2; infantile form; MIM#230900), which is the most severe form, with premature neurological involvement and short survival, and (iii) type 3 (GD3; juvenile form; MIM#231000), which is a mixed form with neurological involvement but with less severe symptoms and a longer life expectancy [7,8].

Symptoms for GD allow us to screen and confirm the disease by the measurement of GCase activity and by variant identification in *GBA1*. Additionally, biomarkers, such as chitriosidase activity, CCL18/PARC concentration, or glucosylsphingosine concentration, are available for diagnosis and for monitoring the response to therapy [9].

Treatment for GD includes the enzymatic replacement therapy (ERT) with imiglucerase, velaglucerase alfa and taliglucerase, increasing the GCase activity, thus leading to substrate clearance. Taliglucerase is not available in the Iberian Peninsula where the study was performed and therefore was not included. Additionally, eliglustat and miglustat are applied to reduce/prevent substrate accumulation without improving the enzymatic function (substrate reduction therapy, SRT). All of these treatments are approved for GD1, while only ERT is available for GD3 and none of them is available for GD2 [10,11,12,13]. Of all these treatments only miglustat is able to cross the blood brain barrier (BBB) and to be biodistributed, with potentially acting on the central nervous system [14]. Still, it remained without any effect on the neurological involvement in GD3 [15].

In addition, *GBA1* variants—pathogenic or non-causative for GD—are recognized as the most prevalent genetic risk factors for sporadic Parkinson’s disease (PD) [15,16], and the risk for developing PD is higher in GD patients, as well as in heterozygous *GBA1*-mutation carriers (GBA-PD), than in non-carriers, suggesting common disease pathways of GD and PD [17]. In the recent years, a great deal of interest has been focused on the relationship between the lipid profile in various tissues and the development of PD [18]. Following this line of investigations, alterations in sphingolipid and—more rarely—phospholipid levels were reported in PD [19,20,21] and also in other neurological diseases, such as Alzheimer’s disease [22] and amyotrophic lateral sclerosis [23].

While most studies reported changes in sphingolipid levels in the blood, less is known about serum phospholipid changes, especially in the context of GD and PD. Thus, here we investigated the serum phospholipid level changes with regards to age, sex, disease severity, and medication in the largest GD and PD cohort from the Spanish Gaucher Registry (FEETEG). 

The main objective of our study was to detect novel associations and novel candidate biomarkers in the largest Spanish GD and PD cohort.

## 2. Results

### 2.1. Clinical and Demographic Data

There was no evidence towards a difference regarding age of onset of PD symptoms (*p* = 0.23), disease duration (*p* = 0.946), or the H&Y score (*p* = 0.581) between the groups (Table 1). A difference appeared between groups in the mean age of blood collection (*p* < 0.001), with GBA-PD as the youngest group. 

### 2.2. Comparison of Absolute Lipid Levels

**Figure 1:**
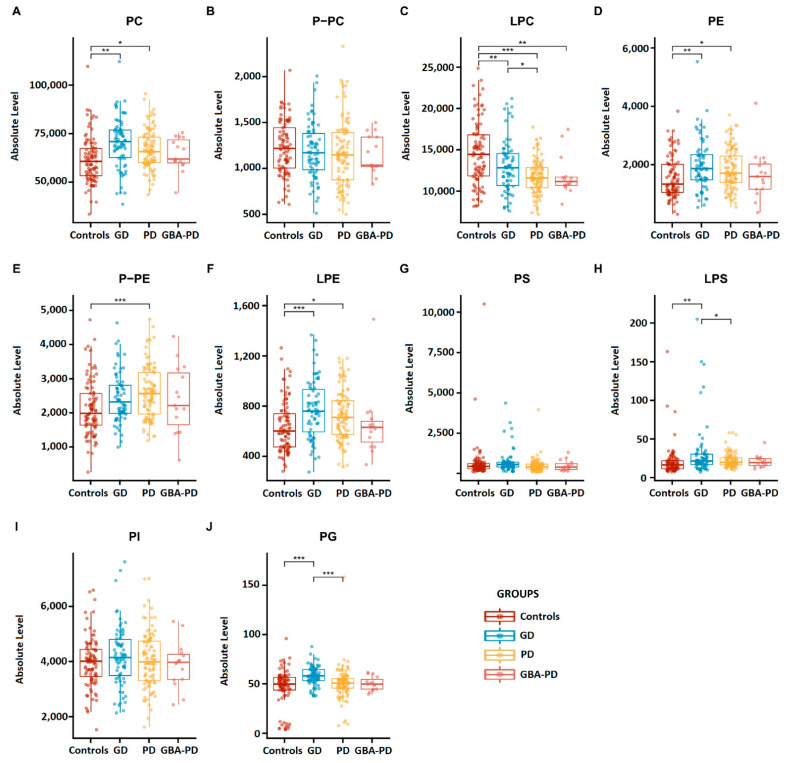
Absolute phospholipid levels plotted by group (**A**–**J**). PD Parkinson’s disease, GBA-PD *GBA1* mutation carrier Parkinson’s, GD Gaucher disease, lipid nomenclature as in the text. Lipid data are expressed as normalized intensities relative to exactly measured internal standards and constitute relative abundances per ml plasma. *, ** and *** denote *p* < 0.05, *p* < 0.01, and *p* < 0.001, respectively.

Absolute lipid levels are shown in Figure 1 and Supplementary Table S1. One-way ANOVAs showed significantly different levels of a range of phospholipid classes between groups (PC: *p* < 10^−4^, LPC: *p* < 10^−8^, PE: *p* = 0.002, LPE: *p* < 10^−3^, P-PE: *p* = 0.001, LPS: *p* = 0.008, PG: *p* < 10^−4^, Supplementary Table S1). Post-hoc tests revealed increased PC, PE and LPE in the GD and PD groups vs. controls, decreased LPC in the GD, PD and GBA-PD groups vs. controls, as well as in PD vs. GD, increased P-PE in PD vs. controls and increased LPS and PG in GD vs. controls and PD. Mean and standard deviation of the absolute lipid levels are shown in Supplementary Table S2.

### 2.3. Multiple Regression Analyses

When including age at sample collection and sex as variables, we found elevated serum PC levels in both the GD and PD patients when compared with controls (*p* < 0.001 and *p* = 0.005, respectively; Table 2A). In contrast, decreased LPC levels were present in these two groups (*p* = 0.001 and *p* < 0.001, respectively) and in the GBA-PD patients (*p* = 0.002) vs. controls (Table 2A). When comparing the three patient groups, the PC and LPC changes had higher magnitudes in the GD than in the PD group (*p* = 0.036 and *p* = 0.008, respectively; Table 2B). The lipid levels were not influenced by the age at blood collection or sex (all *p* > 0.05).

Furthermore, the GD patients showed increased PE (*p* = 0.001), LPE (*p* = 0.0001), and P-PE (*p* = 0.019; Table 2A) levels, when compared with controls. Elevation of LPE (*p* = 0.012) and P-PE (*p* = 0.0004; Table 2A) was also observed in the PD group versus controls. Additionally, the GD patients showed increased levels of LPS and PG (*p* = 0.001 and *p* < 0.001, respectively; Table 2A). 

These results were vastly replicable when assessing lipid levels according to the aforementioned 30% cut-off rate (method #2) and also when analyzing the relative lipid levels (Supplementary Tables S2 and S3).

### 2.4. Associations between Disease Severity, Medication and the Lipid Levels

Dopamine agonist treatment in the PD group showed a positive association with the increase in the PC/LPC ratio and with P-PC levels (*p* = 0.0278 and *p* = 0.014, respectively). On the other hand, the H&Y scale or the disease duration did not influence the lipid levels (Table 2C). All findings were replicable when analyzing relative lipid levels (Supplementary Tables S2 and S3). 

In the GD group, we found that velaglucerase alfa had a positive association with the increased PE and LPE levels (*p* = 0.030 and *p* = 0.002, respectively; Table 2D). On the contrary, miglustat treatment normalized the elevated PC/LPC ratio (*p* = 0.034; Table 2D). The latter finding remained significant when assessing lipid levels according to the aforementioned 30% cut-off rate (method #2) and also when analyzing the relative lipid levels (Table 2D, Supplementary Tables S2 and S3).

## 3. Discussion

Sphingolipids have started to be identified as potential serum biomarkers in various diseases such as hepatitis related to HBV and HCV infection [24], Type I diabetes [25], lysosomal storage diseases [26], Parkinson’s disease [19], and rheumatoid arthritis [27]. Less is known about serum phospholipids in GD, PD and in GBA-PD. Still, an important role of phospholipids in the pathomechanism for GD is plausible, as the disease is characterized by infiltration of phospholipid-laden macrophages (Gaucher cells) in the spleen, liver, bone marrow, lungs and the central nervous system [5,6]. 

Here, we found elevated levels of serum PC with simultaneously decreased levels of LPC, rendering an increased PC/LPC ratio in GD, PD, and GBA-PD patients when compared with the control group. These results were replicable by different analysis methods (#1 and #2) and both when analyzing the absolute or relative phospholipid levels.

This PC/LPC ratio increase was also observed in other studies of PD [28], but—to our knowledge—not yet reported in GD and GBA-PD. Whether this alteration relies on a common pathomechanism in GD and in PD is currently unclear.

The altered PC/LPC ratio also suggests a potential role of the lecithin-cholestrol acyltransferase and/or phospholipase A2 (Lp-PLA2) enzymes, catalysts of the PC-LPC metabolism [29], in GD and PD pathomechanism. In fact, higher serum Lp-PLA2 levels were reported in PD patients when compared to controls [30]. 

In PD patients, we found a positive association of dopamine agonists with the increased PC/LPC and P-PC levels. In previous studies, PC was demonstrated to be entangled in Lewy bodies [31] and also to affect the conformation and aggregation of N-acetylated α-synuclein, which further enhances binding to micelles rich in PC [32]. While decreased levels of some PC species and increased levels of others were found in the brains [33] and in the plasma [34] of PD patients, an association of dopamine agonists with such PC and LPC changes—to our knowledge—has not been reported yet. The fact that the results were not replicable in the 30% cut-off analysis, instead showing an association of dopamine agonists with a decrease in PC levels (Supplementary Table S3), might suggest that this medication might differentially impact individual PC species in serum. Indeed, non-detectable species might be the result of a technical error or a true reflection of very low or absent expression of certain types of PC. Future research aimed at analyzing lipid species will help in clarifying this hypothesis. 

Dopamine has been shown to increase PC production in rat synaptosomes, an effect blocked by haloperidol, a dopamine antagonist, suggesting modulation through dopamine receptors [35]. Furthermore, dopamine agonists might increase phospholipase D activity [36,37], an enzyme which converts PC into phosphatidic acid and choline and has been associated with a variety of neurodegenerative diseases [38]. Altogether, these results suggest that dopamine might activate PC-related cellular cascades, which may be altered in PD or affected by PD medication. 

On the other hand, LPC was shown to be increased in the brain of PD rodent models and acting to decrease dopamine receptor 1 and 2 sensitivities in the striatum [39]. Moreover, injection of LPC into the brain ventricles in rats was shown to decrease dopamine turnover and reproduce Parkinson-like behaviors [40]. As mentioned above, the phospholipase A2 family can convert PC into LPC, thus providing a possible mechanistic link for our findings. These enzymes might play a role in inflammatory cascades and neurodegeneration [41,42] and missense mutations to one of these enzymes has been linked to a form of PD [43]. Our findings of increased PC/LPC ratio in the serum of PD patients treated with dopamine agonists prompt further research into a putative lipid modulation by dopamine and its disruption in PD. 

Furthermore, we also found elevated levels of LPE and P-PE in our PD patients. Comparatively, decreased levels of PEs were reported in the brains of early PD patients [44] and one study found reduced plasma levels of P-PEs in PD [45]. Importantly, we did not find significant differences between PD and GBA-PD in the measured serum lipids. This might be due to the lack of specificity of these lipids in signaling changes in the pathophysiology of these two groups or a consequence of a smaller sample size of GBA-PD carriers. Future work is needed to clarify whether serum lipid levels can differentiate these two groups. 

Regarding the GD patients, similar to our observations in the serum, increased levels of PC, PI and PG have been reported in fibroblast extracts of GD1 and GD2 patients [46]. Elevated PC synthesis has also been reported in neuronal cells in a mouse model of GD [47], and in this model the increase was reported to be due to the activation of phosphocholine cytidylyltransferase, which is the rate-limiting enzyme step in PC synthesis [48,49]. A common pathophysiologic pathway in serum, fibroblasts and neurons might be present. 

Moreover, a study of Meilke et al. found differences in the levels of some PC and PI species in the serum of GD patients vs. controls [50]. In this study, GD patients under ERT treatment showed similar increase in the phospholipid species, as those without ERT, suggesting that ERT has no preventive effect on the phospholipid changes in GD. Compatibly, in our study, GD patients showed increase in PE and in LPE, and a positive association between ERT with velaglucerase alfa and the increased PE and LPE levels was demonstrable. 

On the contrary, we found in these patients a normalizing effect of miglustat on the PC and LPC changes, and this finding was replicable with all methods.

Miglustat is a small iminosugar, a reversible inhibitor of glucosylceramide synthase, approved for GD treatment, as well as for other lysosomal storage diseases such as Niemann-Pick type C [51]. Miglustat acts in the central nervous system, where it improves and stabilizes neurological functions [52]. One of the hypothesized effects of miglustat is restoring lipid trafficking, although this mechanism is still not completely understood. Miglustat appeared to have beneficial effects on plasma lipid, lipoprotein, and C-reactive protein concentrations in therapy-naïve GD1 patients, resulting in an improved atherogenic lipid profile [53]. Furthermore, in Niemann-Pick type C, the depletion of glycosphingolipids by miglustat treatment was found to reduce pathological lipid storage, improve endosomal uptake and normalise lipid trafficking in peripheral blood B lymphocytes [54]. Such mechanism might also explain the normalizing effect of miglustat on the PC/LPC changes that we found in our GD patients.

## 4. Materials and Methods

### 4.1. Patients

**Figure 2:**
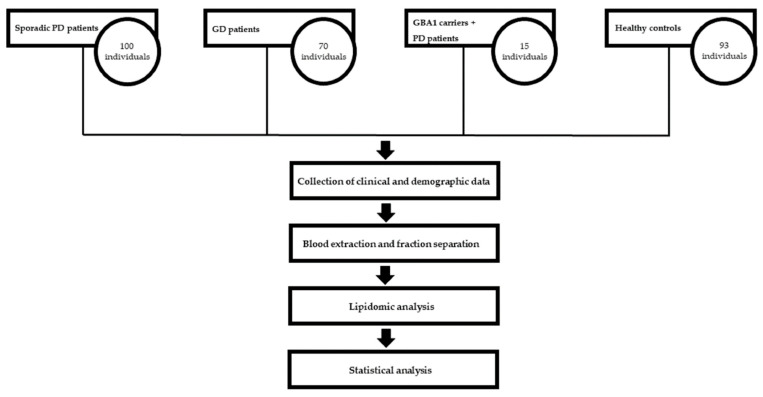
Study diagram.

We collected clinical, demographic, and laboratory data from 278 participants in total: 100 sporadic PD patients (variants in *GBA1* were excluded), 70 GD patients (GD1 and GD3 patients with confirmed bi-allelic *GBA1* pathogenic variants), 15 heterozygous *GBA1*-variant carriers who developed PD (GBA-PD), and 93 healthy controls (Figure 2).

The sample sizes of the groups were comparable to those used in previous articles in which similar methods were applied [19,27], and they were shown to be sufficient in detecting significant differences between groups (*p* < 0.05, uncorrected) with a power of 0.8, provided that the effect size was large. Power analyses were conducted using both reference lipid values from previous studies and assuming a Cohen’s *d* ≥ 1.2. All between-group size ratios (e.g., 100/93 or 15/100) were tested, and all analyses were performed using G*Power 3.1.9.7 (University of Düsseldorf, Düsseldorf, Germany) [55,56].

All participants were >18 years of age and signed bioethical informed consent authorizing the use of their biological samples for research purposes was obtained. The study was approved by the Ethics Committee of the University of Aragón (Spain) and by the Ethics Committee of the University of Lisbon.

GCase activity was measured in 58 GD, 4 GBA-PD patients, and 6 controls but not in PD patients as in this group the blood samples were not enough to obtain leukocytes (Table 1). The GCase measurement was conducted according to previously published fluorometric methods using 4-methylumbelliferyl-b-D-glucopiranoside (Sigma-Aldrich-Merck, Madrid, Spain) as a substrate [57]. Data on therapy in the GD group with ERT (imiglucerase or velaglucerase alfa) or with SRT (eliglustat or miglustat) was available in 69 patients (42 treated and 27 non-treated). The Hoehn and Yahr (H&Y) scale was collected from 46 PD and 5 GBA-PD patients. Data on PD therapy with dopamine agonists was obtained from 82 PD and from 10 GBA-PD patients, while therapy with other antiparkinsonian drugs was collected from 44 PD and 6 GBA-PD patients.

### 4.2. Quantification of the Levels of Lipid Groups and Their Species

Levels of phosphatidylcholine (PC), lyso-phosphatidylcholine (LPC), plasmalogen-phosphatidylcholine (PLC), phosphatidyl-ethanolamine (PE), lyso-phosphatidyl-ethanolamine (LPE), plasmalogen-phosphatidyl-ethanolamine (PPE), phosphatidylserine (PS), lyso-phosphatidylserine (LPS), phosphatidylinosiltol (PI), and phosphatidylglycerol (PG) were measured using high-performance liquid chromatography–mass spectrometry (HLPC-MS).

Lipid levels were quantified in two distinct manners: (method #1) by summing the levels of all species (considering all non-detectable values to be null) [19]; and (method #2) by summing the levels of the species that had less than 30% non-detectable values across the full sample (i.e., the sample including PD, GBA-PD, GD patients, and controls). Such cut-off was used to analyze whether the number of non-detectable values influenced the results. All lipid levels were assessed as absolute (i.e., measured) as well as relative (i.e., percentage of the total phospholipid) values.

### 4.3. Statistics

#### 4.3.1. Comparison of Clinical Data and Absolute Lipid Levels

Sex prevalence was compared between groups using a Chi-squared test. Age of symptom onset of PD, disease duration, and H&Y score at blood collection were compared between groups (PD vs. GBA-PD) using *t*-tests. Age of blood collection, GCase activity, and the absolute lipid levels were compared between the applicable groups using one-way ANOVAs, followed by TukeyHSD in statistically significant comparisons.

#### 4.3.2. Between-Group Analyses of Lipid Levels

Lipid levels were analysed between groups using multiple-regression models (in addition to the aforementioned ANOVAs), in which each lipid level of interest (quantified by summing the levels of the respective species; section “Quantification of the levels of lipids and their species”, method #1) was used as the dependent variable and the subject’s group was included as an independent categorical variable (reference category: control subjects; other categories: PD, GBA-PD, GD patients). In each model, two other independent variables were included: age at sample collection and sex, as these are the most important demographic confounds that should and are mostly accounted for in lipidomic analyses [19,22]. Contrast tests using F-test statistics were performed to compare coefficients between groups. 

All models were replicated using the lipid levels quantified through the sum of the levels of the respective species with few missing values (section “Quantification of the levels of lipids and their species”, method #2). 

#### 4.3.3. Within-Group Analyses of Lipid Levels

Lipid levels were also analyzed within groups using multiple-regression models, in which each lipid level of interest (method #1 for the main analyses; method #2 for the confirmatory, supplementary analyses) was used as the dependent variable. 

In the PD group analyses, H&Y scale, disease duration, and PD medication (dopamine agonists and antiparkinsonian drugs) were included, besides age at sample collection and sex, as additional independent variables. In parallel, in the GD group, we performed multiple regressions including medication (coded categorically for imiglucerase, velaglucerase alfa, eliglustat, or miglustat, with the reference category being “no medication”), in addition to age at sample collection and sex, as independent variables.

## 5. Conclusions

Supporting the robustness of our findings, the majority of the results remained significant when summing the levels of exclusively those lipid species with less than 30% non-detectable values across the full sample (method #2, see above) and when using the relative lipid levels. Such control analyses are desirable in lipidomics analyses, as the phospholipid concentrations in the blood are a priori very low and the sensitivity of the array might not always detect reduced or severely reduced levels of some lipid species.

Taken together, we described significant phospholipid level changes in the serum of GD, GBA-PD and PD patients, which may suggest a role of such lipids in the pathomechanism of these diseases. We demonstrated, for the first time, the normalizing effect of miglustat treatment on such phospholipid changes in GD.

Furthermore, our results might help to detect novel early PD biomarkers by comparing the serum lipid level changes with other PD biomarkers, such as SPECT-Scan, to evaluate their sensitivity in the early disease detection and their value in the differential diagnosis of the disease.

### Limitations of the Study

It should be noted that the limited number of patients available for a rare disease such as GD makes it necessary to interpret the results with caution. The sub-distribution by treatment groups in the GD patients represents a small sample number that requires validation in a larger number of patients to avoid bias and misinterpretation. The observational nature of our study does not allow us to draw conclusive causal links in our findings. Moreover, the relationship between serum lipid levels and pathophysiological mechanisms needs elucidation by experimental studies. Further studies on other biochemical data for both GD and PD groups and lipid levels might be interesting to be analyzed. 

Furthermore, we did not correct our statistical analyses for multiple comparisons. While we opted not to do so to minimize the risk of Type II errors, such approach leads necessarily to an increased risk of Type I errors. Future studies with larger samples, or stronger a priori hypotheses, possibly based on our results, are thus necessary to further validate our findings.

## Figures and Tables

**Table 1:** Clinical, demographic and therapy data of the patient and control groups.

	PD	GBA-PD	GD	Controls	*p*-Value
**N (total = 278)**	100	15	70	93	-
**Demographic and Clinical Data:**					
**Male (%)**	60 (60%)	7 (50%)	27 (38.6%)	36 (39.4%)	X2 (3) = 11.34; *p* = 0.01
**Mean age of symptom onset (years) (± std)** **PD (n = 100)** **GBA-PD (n = 11)**	59.97 (±10.09)	56 (±12.88)	-	-	t (109) = 1.2041, *p* = 0.23
**Mean age of blood collection (years)** **(±std)**	68.0 (±8.62)	58.3 (±13.99)	67.5 (±7.27)	63.0 (±15.49)	F (3, 274) = 5.75, *p* = 0.001
**Mean Disease Duration (years) (±std)** **PD (n = 100)** **GBA-PD (n = 11)**	8.04 (±5.90)	7.91 (±8.05)	-	-	t (109) = 0.067, *p* = 0.946
**Mean GCase activity (nmol/mg/h) (±std)** **GD (n = 58)** **GBA-PD (n = 4)** **controls (n = 6)**	-	5.38 (±1.95)	0.98 (±0.68)	6.15 (±1.66)	F (2,65) = 127.5, *p* < 0.001
**Mean Hoehn and Yahr score at blood collection (±std)** **PD (n = 46)** **GBA-PD (n = 5)**	3.26 (±1.02)	3.00 (±0.71)	-	-	t (49) = 0.55, *p* = 0.581
**Patients treated with Antiparkinsonians (%)**	44 (44%)	6 (40%)	-	-	X2 (1) = 0.0001; *p* = 0.9903
**Patients treated with Dopamine Agonist (%)**	82 (82%)	10 (66.7%)			X2 (1) = 1.0781; *p* = 0.299
**Patients treated with imiglucerase (%)**			12 (17%)		
**Patients treated with velaglucerase alfa (%)**			16 (23%)		
**Patients treated with eliglustat (%)**			6 (9%)		
**Patients treated with miglustat (%)**			8 (12%)		

PD Parkinson’s disease, GBA-PD GBA mutation carrier Parkinson’s, GD Gaucher disease.

**Table 2:** Serum lipid level alterations in the Iberian GD and PD cohort.

**Phospholipids**	**PC**	**LPC**	**PC/LPC**	**P-PC**	**PE**	**LPE**	**PE/LPE**	**P-PE**	**PS**	**LPS**	**PS/LPS**	**PI**	**PG**
**Between-Group Analyses**													
**A. Patient Groups vs. Control Group**													
**Intercept**	0.532 ***	0.560 ***	4.408 ***	0.435 ***	0.149 ***	0.363 ***	0.467 ***	0.389 ***	0.068 *	0.099 ***	0.611 ***	0.460 ***	0.289 ***
**PD (n = 100)**	0.043 **	−0.114 ***	1.268 ***	−0.036	0.035	0.052 *	0.009	0.086 ***	−0.018	0.005	−0.209 ***	0.009	0.023
**GBA-PD (n = 15)**	0.025	−0.105 **	1.085 ***	−0.026	0.026	0.012	0.032	0.056	−0.016	0.003	−0.166	−0.005	0.022
**GD (n = 70)**	0.076 ***	−0.063 **	1.131 ***	−0.021	0.070 **	0.088 ***	0.030	0.061 *	0.009	0.056 **	−0.092	0.025	0.073 ***
**Sex**	0.011	0.002	0.019	0.02	0.029	0.018	0.039	0.03	0.012	0.019	0.030	−0.009	0.012
**Age of Collection**	0.018	0.033	0.128	0.116	0.169 ***	0.081	0.250 *	0.068	−0.017	−0.01	0.016	0.089	−0.0003
**B. Between Patient Groups**													
**PD vs. GD**	−0.033 *	−0.051 **	0.183	−0.015	−0.035	−0.036	−0.021	0.025	−0.027 *	−0.051 **	−0.117	−0.016	−0.05 ***
**PD vs. GBA-PD**	0.018	−0.009	0.137	−0.01	0.009	0.04	−0.023	0.03	0.002	0.002	−0.043	0.014	0.001
**GD vs. GBA-PD**	0.051	0.042	0.046	0.005	0.044	0.062	−0.002	0.005	0.025	0.053	0.074	0.03	0.051
**Within-Group Analyses**													
**C. Associations with PD Disease Severity, Medication and Disease Duration (*n* = 46; PD patients)**													
**Intercept**	0.752 ***	0.733 ***	4.032 ***	0.203 ***	0.338 *	0.732 ***	0.358	0.693 **	0.132	0.136 *	1.196 *	1.006 ***	0.509 **
**Hoehn and Yahr scale**	−0.092	−0.087	0.212	0.073	−0.071	−0.094	−0.047	0.087	−0.029	0.009	−0.311	−0.162	−0.128
**Dopamine Agonist**	0.044	−0.027	0.870 *	0.139 *	0.019	−0.025	0.077	0.113	0.02	0.008	0.073	−0.005	0.083
**Anti-Parkinsonian**	−0.025	−0.05	0.437	0.0009	−0.033	−0.068	0.027	−0.016	0.015	−0.026	0.186	−0.031	0.069
**Sex**	−0.045 *	−0.014	−0.373	0.024	−0.045	−0.043	−0.057	−0.031	−0.002	−0.006	0.013	−0.114 **	−0.004
**Age of Collection**	−0.133	−0.169	0.679	0.19	0.089	−0.13	0.364	−0.29	−0.11	−0.031	−0.874	−0.363 *	−0.206
**Disease Duration**	−0.06	−0.097	0.830	−0.176	−0.006	−0.082	0.258	−0.185	−0.022	0.004	−0.153	−0.089	−0.172
**D. Associations with GD Therapy [on Therapy (n = 42) vs. Untreated (n = 27) GD Patients]**													
**Intercept**	0.516 ***	0.451 *	6.221 ***	0.509 **	−0.072	0.270	0.165	0.342	−0.021	0.199	−0.520	0.343	0.602 ***
**Imiglucerase (n = 12)**	0.020	−0.057	0.418	0.034	−0.035	0.026	−0.0688	−0.047	0.031	−0.008	0.012	−0.018	0.028
**Velaglucerase alfa (n = 16)**	0.064	0.012	0.165	0.097	0.094 *	0.154 **	−0.039	0.007	0.034	0.033	−0.030	0.029	0.056
**Eliglustat (n = 6)**	0.094	−0.008	0.443	0.138 *	0.053	0.050	0.018	0.119	−0.026	−0.072	0.431	0.097	0.147 **
**Miglustat (n = 8)**	−0.032	0.099	−1.133 *	−0.033	−0.072	0.019	−0.172	−0.039	−0.036	0.031	−0.406	−0.024	−0.050
**Sex**	0.035	0.079 *	−0.323	−0.016	0.098 **	0.062	0.095	0.087*	0.035	0.040	0.307	0.060	0.022
**Age of Collection**	0.094	0.164	−0.611	0.068	0.497 *	0.290	0.631	0.199	0.219	−0.085	2.266	0.229	0.035

(**A**). Patient groups compared with the control group. Results obtained, when controlling for sex and age at blood collection. Patients’ sex coded as 0 (female) or 1 (male). (**B**). Patient groups compared with each other. (**C**). Association of PD disease severity, therapy and disease duration with lipid level changes. (**D**). Association of GD therapy with lipid level changes. (**A**–**D**). Results are presented as analyzed by method #1 (see “Quantification of the levels of lipid groups and their species”). Lipid nomenclature as in the text. Effects of the independent variables on the normalized lipid levels (dependent variables) are expressed by the corresponding estimated regression coefficients, with positive (negative, respectively) coefficients meaning that higher values of the independent variables are associated with higher (lower, respectively) values of the lipid levels. All variables were scaled so that their maximum absolute value was equal to 1. *, ** and *** denote *p* < 0.05, *p* < 0.01, and *p* < 0.001, respectively. Coefficients with P-values below <0.05/3 (i.e., surviving Bonferroni correction) are shown in bold.

## Supplementary Materials

The following supporting information can be downloaded at: https://www.mdpi.com/article/10.3390/ijms231810387/s1.
