# Supplementary material for: Serum Phospholipid Profile Changes in Gaucher Disease and Parkinson’s Disease
Source: Int J Mol Sci. 2022 Sep 8;23(18):10387. doi: 10.3390/ijms231810387 (PMC9499334; doi:10.3390/ijms231810387)
Supplement: Supplementary file 1 [file ijms-23-10387-s001.zip › ijms-1870746-supplementary.pdf]

Supplementary Table S1.

Serum lipid level alterations in the Iberian GD and PD cohort. Absolute lipid levels. Lipid data are expressed as normalized intensities relative to exactly measured internal standards and constitute relative abundances per ml plasma. Sample number 1-100: PD, 101-119: GBA-PD, 132-231: controls, 232-301: GD

Sample number	PC	P-PC	LPC	PE	P-PE	LPE	PS	LPS	PI	PG	Total PL
1	59592.7	500.4	10927.3	1718.1	1790.5	506.0	206.6	17.5	4934.5	42.7	80236.3
2	54147.5	657.9	7184.0	1746.6	1443.4	316.0	434.1	16.4	3281.6	36.3	69263.8
3	59452.0	549.7	12598.7	2438.9	2088.9	922.8	341.5	20.1	3949.4	31.8	82393.7
4	61894.4	815.3	11030.0	1793.4	2705.4	734.9	365.2	19.7	4892.5	7.7	84258.7
5	60124.4	645.3	11373.3	759.5	2650.3	604.6	269.3	24.3	3239.0	35.0	79724.9
6	59922.6	1160.3	13092.1	1164.6	1509.2	658.0	500.9	16.3	3352.7	45.6	81422.5
7	55499.6	797.3	10017.0	1648.8	1730.3	696.5	435.3	15.2	3912.9	32.6	74785.5
8	53967.7	1175.4	10591.7	1560.2	2487.9	564.4	115.1	14.0	5303.7	43.3	75823.4
9	65923.9	1162.8	12196.5	1718.7	1796.6	884.8	214.7	21.2	4392.1	42.0	88353.4
10	52781.7	674.4	12181.1	1627.9	2073.0	1004.4	288.1	15.1	3223.4	33.5	73902.7
11	43444.9	876.2	10415.7	838.2	1321.3	576.4	170.0	15.0	2245.2	36.2	59939.3
12	59149.2	797.7	9374.9	2579.9	1749.6	625.3	446.4	10.8	3912.6	41.5	78687.9
13	67039.4	691.0	13388.8	1665.4	2325.1	817.4	125.7	26.9	3134.2	52.7	89266.6
14	65807.3	1539.3	11970.7	2196.4	3114.0	746.3	889.5	31.2	4215.5	39.0	90549.1
15	60043.8	1003.3	12614.4	1767.9	2456.2	696.6	269.3	13.6	2565.1	51.2	81481.4
16	67038.8	1244.4	11680.4	2899.8	2987.8	816.3	586.9	14.6	2419.4	9.3	89697.7
17	66315.1	686.2	13527.7	2287.2	2264.0	763.2	500.5	19.9	3818.9	44.7	90227.4
18	79086.3	775.5	9432.1	993.2	4130.4	327.9	3952.1	26.9	3052.1	158.4	101934.9
19	65582.2	884.9	10611.0	976.9	1964.9	494.2	560.0	19.6	3495.6	50.0	84639.2
20	62553.5	1901.0	12234.9	1888.4	4147.5	780.0	118.2	23.5	4087.8	45.0	87779.7

21	75024.2	1224.8	13119.4	1394.6	2531.0	642.2	103.4	18.2	4944.1	53.1	99055.1
22	56805.9	1146.0	11290.4	1048.0	2568.3	632.7	277.8	15.7	3455.2	40.5	77280.6
23	69615.7	1926.4	13599.3	2528.5	4097.6	834.5	250.9	11.9	4533.7	53.5	97452.0
24	73642.4	1288.8	12816.3	860.4	2551.8	646.5	583.6	29.4	4108.3	59.1	96586.5
25	65875.1	1289.7	10871.2	2063.7	2403.1	565.6	316.4	26.9	2784.0	49.3	86245.1
26	63887.1	1164.8	11985.6	1224.4	3412.3	699.4	79.7	20.3	3250.4	52.4	85776.3
27	70964.8	1080.8	12849.5	1681.6	3257.1	696.2	303.8	25.5	4841.8	54.7	95755.7
28	59287.8	1113.0	9827.6	1449.1	2797.0	574.6	126.4	12.9	3295.7	41.6	78525.8
29	61503.4	1373.7	11488.9	2036.4	3323.4	935.2	404.8	21.0	3557.6	47.4	84691.7
30	95543.5	1415.4	15731.9	3194.8	3432.2	1045.1	349.1	18.8	7015.3	48.7	127794.9
31	51232.5	710.4	10556.9	672.7	1817.4	433.8	464.0	18.3	1622.1	37.5	67565.6
32	65531.1	814.7	13122.9	1391.4	1712.6	1025.4	385.8	16.8	2616.0	45.7	86662.5
33	65801.7	1131.3	10304.0	1613.2	2706.5	526.3	296.1	22.7	1935.8	57.4	84395.1
34	77528.6	1167.2	13933.1	1969.0	3440.1	731.5	394.4	18.8	5606.5	57.5	104846.6
35	60531.1	876.8	13056.5	1303.7	4740.4	772.0	545.4	16.4	3219.4	46.0	85107.8
36	78003.3	1092.7	11501.9	1629.3	1853.8	710.0	200.1	22.0	5577.9	57.8	100648.7
37	66545.4	815.0	11235.4	2140.7	2651.5	810.6	987.7	46.0	3467.8	45.6	88745.6
38	59138.2	909.2	11458.1	914.1	1953.8	677.2	524.2	14.4	6218.6	45.9	81853.7
39	48777.5	624.5	11566.7	1751.1	2592.8	893.2	506.8	20.4	3735.9	27.7	70496.7
40	73074.1	1330.0	10630.6	2332.3	2186.4	670.5	490.0	16.0	4386.9	56.8	95173.6
41	58858.0	751.8	12727.2	1423.7	2687.1	439.2	377.1	16.5	2581.7	50.3	79912.6
42	62893.5	1105.1	11404.9	1710.5	1805.4	702.0	675.6	19.1	3716.1	52.9	84085.1
43	64325.4	998.1	11693.9	1013.9	1972.3	558.7	578.3	18.9	3091.5	50.9	84301.9
44	82794.0	1550.4	12250.8	3138.9	2362.9	823.0	747.3	21.2	4739.4	64.8	108492.8
45	72380.3	1063.4	13699.1	2753.5	3645.1	1074.4	279.6	23.9	3915.6	12.6	98847.5
46	60808.7	736.6	10413.7	1133.6	2903.1	521.9	526.4	17.0	2734.4	49.9	79845.3
47	50863.3	1149.4	7739.3	546.0	1181.8	337.3	330.3	14.5	2818.0	44.2	65024.2
48	56520.9	1048.7	15054.6	2674.0	2621.3	875.9	474.3	17.0	4324.3	50.4	83661.4
49	69007.4	1086.9	9857.9	1998.9	2885.5	547.2	165.4	17.0	5147.4	54.0	90767.6
50	45955.7	750.2	8739.0	1415.7	1728.5	615.5	294.4	24.7	3518.8	36.8	63079.3
51	74033.6	874.0	12634.5	1908.6	3319.3	892.1	247.3	12.7	5118.0	53.0	99092.9

52	74868.7	1121.9	12176.9	2497.7	3321.5	1181.3	144.2	19.4	4990.9	60.2	100382.7
53	68366.8	1125.3	10325.4	2596.6	3208.5	568.0	414.0	19.7	3977.9	59.3	90661.5
54	55723.8	1122.0	9381.3	2628.2	1674.6	716.3	251.3	27.7	3768.2	47.9	75341.3
55	53380.2	1156.5	12152.4	2550.8	2196.9	1035.0	729.1	17.5	4255.3	54.8	77528.5
56	60376.3	848.0	8274.6	1658.3	2562.8	550.4	638.7	25.1	3306.9	47.8	78288.9
57	65393.1	1412.8	11519.0	1976.3	3299.1	757.2	285.1	17.5	3932.3	67.1	88659.5
58	68441.3	1197.0	10014.2	3348.7	2418.6	655.5	143.6	19.8	2851.8	67.2	89157.7
59	53441.1	716.5	11586.4	1391.5	2653.4	1048.3	439.6	33.0	3291.7	36.8	74638.2
60	61257.0	1595.1	8699.3	1520.3	1781.2	551.0	438.5	21.1	3754.6	55.8	79673.8
61	60076.4	1002.6	11832.6	3067.8	3209.3	1181.1	623.7	34.8	4739.9	52.5	85820.6
62	59683.4	961.4	7984.9	1478.4	1879.8	605.7	107.7	18.5	3191.0	46.0	75956.9
63	60912.3	1406.4	11679.0	2419.4	2342.5	639.7	577.6	28.9	4689.0	54.4	84749.1
64	65576.9	1300.1	10909.2	1449.8	2499.5	557.7	843.4	27.0	4308.2	50.6	87522.6
65	69115.9	839.3	15215.2	2211.3	3617.5	1050.7	382.2	22.6	5588.1	63.4	98106.1
66	80521.0	1383.4	12851.0	2079.8	3727.1	1042.7	572.8	35.3	4530.5	64.1	106807.6
67	70040.0	1343.1	10889.0	1703.9	2349.0	671.2	759.9	26.3	4683.7	58.0	92524.1
68	69772.1	1569.9	11236.6	3344.8	1708.2	994.1	377.6	33.6	4561.5	11.0	93609.3
69	61180.7	1892.6	9913.5	1622.5	1858.4	606.4	266.2	17.9	3971.1	49.5	81378.7
70	57593.5	1055.8	11219.7	1405.7	1367.1	894.9	1130.7	58.1	2979.6	50.6	77755.5
71	72054.3	1143.0	12659.6	1518.5	3923.1	842.9	415.4	55.4	5024.2	51.0	97687.4
72	78277.6	1262.9	9351.3	1645.3	2717.8	466.5	250.6	15.9	4729.7	55.4	98773.1
73	84391.2	1220.7	12099.9	1377.5	3337.8	553.5	581.7	19.2	5933.4	63.9	109578.9
74	87510.9	1945.9	15505.0	1191.2	2588.8	588.0	631.7	23.0	4191.4	69.5	114245.5
75	58550.2	747.6	9421.2	1707.0	1667.5	748.7	460.3	35.9	2897.7	54.8	76290.9
76	60804.5	734.4	9760.8	1047.9	2249.4	499.0	391.3	15.0	3924.7	46.7	79473.6
77	76632.8	1776.7	11219.4	2124.2	3301.7	873.5	703.3	25.8	3998.6	54.1	100710.2
78	68269.5	968.9	9154.7	2711.2	3407.6	737.3	637.2	21.6	4071.0	46.9	90026.1
79	84089.4	869.9	11835.1	2794.7	3169.3	857.6	632.8	17.1	5603.4	63.4	109932.6
80	79459.2	1049.8	11583.8	1861.1	2923.5	738.4	345.7	17.4	4905.5	64.9	102949.4
81	77571.0	1433.3	14275.4	883.6	2788.5	794.7	168.4	9.5	4310.5	54.7	102289.6
82	86006.9	1179.7	13696.3	2527.2	4210.8	839.6	84.6	18.9	4872.7	74.4	113511.0

83	73547.1	1781.6	13197.4	3706.9	2603.5	1091.2	205.2	10.5	4807.8	65.1	101016.3
84	59287.4	1511.3	7379.2	1904.4	1305.3	452.7	986.8	21.8	3447.5	45.8	76342.3
85	74734.6	1423.6	11032.8	2710.0	2350.1	845.5	213.0	20.0	4227.8	55.6	97612.9
86	66653.1	1493.2	12194.7	1287.5	2093.5	718.9	864.8	20.8	4131.9	53.0	89511.3
87	60303.9	1305.4	9922.1	1157.2	2570.5	506.6	310.9	17.3	2688.3	51.0	78833.0
88	70741.3	1416.5	11418.7	1459.9	2302.9	570.1	345.0	24.5	4771.6	54.1	93104.6
89	68722.5	1155.9	14481.1	3211.5	2810.8	1102.4	522.2	13.1	4214.5	53.0	96287.0
90	61719.5	1260.5	8836.3	1105.8	1763.3	501.4	326.0	15.8	4294.9	52.7	79876.2
91	81395.9	1413.4	16398.7	1537.7	1813.9	800.1	340.9	13.0	6020.0	68.3	109802.0
92	64379.6	1407.3	10237.8	1210.6	2183.1	479.0	488.9	11.5	3918.8	53.8	84370.3
93	92661.5	1820.0	11707.6	2208.8	2714.9	694.2	1334.9	35.0	6997.5	58.9	120233.4
94	64795.5	1226.0	13263.5	2267.4	2836.8	820.4	411.1	27.5	3690.1	46.3	89384.6
95	71365.7	1170.2	14094.4	1894.1	3235.0	718.7	778.7	26.2	4792.0	49.1	98124.0
96	84823.2	2328.6	15664.4	2499.6	4519.6	933.3	403.5	31.0	4068.9	72.7	115344.7
97	76744.9	1506.0	17754.4	1505.5	2681.4	1151.7	581.5	57.9	4416.5	50.6	106450.3
98	66446.0	1599.5	16251.5	969.1	3459.4	739.4	665.6	38.3	3913.8	50.4	94133.0
99	62258.5	1089.2	12564.6	798.4	1735.0	625.3	382.1	12.7	3667.3	47.2	83180.3
100	77493.6	1959.1	13541.1	2636.9	2394.4	711.7	501.8	35.0	5107.9	54.2	104435.9
101	60576.3	979.4	10832.9	1167.9	2213.6	474.8	379.9	14.5	3924.1	48.7	80612.2
102	60684.6	1339.3	11639.0	1378.7	3681.6	595.6	184.0	11.2	3715.6	43.4	83273.0
103	59284.9	887.1	10073.5	1708.9	1393.4	551.0	278.4	18.9	3190.0	56.8	77442.9
104	75504.8	1343.4	10831.7	1593.9	1872.7	648.7	144.4	22.5	5305.1	49.7	97316.8
105	61010.5	1422.0	10490.2	1484.6	1428.1	657.6	1301.6	45.2	3969.5	48.9	81858.1
106	58857.9	1182.3	10725.1	1740.1	4235.6	748.7	141.8	19.4	4439.1	44.6	82134.6
107	68179.1	1034.7	10681.3	2236.2	2114.3	637.3	392.1	26.3	4042.0	50.6	89393.9
108	70469.0	1240.3	11581.9	1964.4	2470.2	630.6	446.1	24.5	5455.4	61.4	94343.9
109	73731.3	1026.9	11475.0	2262.5	3285.8	619.2	682.2	24.6	4022.9	60.1	97190.6
110	73068.4	1417.0	14087.5	4104.6	2599.6	1491.5	202.5	23.4	4312.8	52.1	101359.3
111	55511.2	1031.8	8398.2	692.3	1422.0	333.9	855.1	17.2	2434.9	41.2	70737.9
113	67083.9	1014.3	11163.5	1153.8	3051.0	475.6	966.0	14.1	2612.7	51.6	87586.3
115	61892.9	1499.1	11753.9	1007.5	2116.8	441.1	542.7	16.5	3328.8	44.7	82643.9

118	73354.6	1027.4	17466.5	2081.1	3353.3	758.0	543.3	27.2	4206.4	60.6	102878.4
119	44503.4	829.1	16649.4	360.8	609.4	699.3	276.0	13.7	3353.6	39.4	67334.2
132	54920.7	932.0	10774.3	1100.7	1876.4	457.3	601.3	12.3	3185.5	44.8	73905.3
133	53668.0	1076.8	20469.2	1077.9	2331.0	739.4	498.9	22.8	3699.0	40.9	83623.9
134	55327.4	1235.6	15944.3	1924.9	2060.5	595.1	988.5	17.9	3921.3	10.9	82026.3
135	51739.4	1506.7	21146.0	801.2	1295.0	642.5	507.3	18.3	4398.4	4.8	82059.7
136	51378.7	756.6	9001.0	1163.7	1846.2	317.3	710.5	10.8	2309.4	45.4	67539.5
137	61581.8	1397.7	8133.7	761.9	1770.5	333.3	211.6	13.7	3212.5	51.9	77468.6
138	63429.5	952.5	9975.4	1846.9	3129.3	433.1	381.8	9.0	4639.5	56.4	84853.3
139	33396.1	1139.3	8202.4	392.9	259.3	469.7	362.0	21.3	1523.9	42.7	45809.6
140	54047.4	1368.8	17465.0	1002.0	2216.0	470.6	463.2	11.8	4065.9	52.2	81162.9
141	62751.8	1235.4	16445.5	1031.1	1983.9	525.9	1381.8	23.5	4465.2	48.8	89892.9
142	66778.9	1530.0	15649.9	1771.2	1038.5	858.8	277.7	11.2	5151.0	8.4	93075.6
143	51564.2	1058.9	11426.2	299.6	1603.1	280.0	566.7	11.1	2591.7	38.2	69439.8
144	60673.7	918.0	12588.8	1071.0	1792.5	526.7	394.8	17.0	3324.9	43.5	81350.9
145	57070.1	629.3	18749.8	1651.8	2107.2	763.6	228.9	18.7	3885.0	47.6	85152.1
146	78618.8	1722.3	11402.7	662.0	2309.0	408.6	10506.7	163.2	4020.7	63.2	109877.2
147	50782.1	1087.3	19843.6	1133.1	1805.4	618.0	420.8	18.7	4697.9	49.4	80456.3
148	57142.9	1512.7	15421.6	2159.8	839.8	681.4	299.7	24.8	4090.5	54.8	82228.0
149	64761.5	1531.1	14528.0	1726.9	2231.9	647.4	559.5	16.7	4071.7	53.6	90128.3
150	71943.4	1530.2	11701.1	1274.7	2129.1	442.5	763.9	15.4	4392.6	7.3	94200.3
151	56153.8	1095.2	14486.0	1359.7	1408.4	582.1	528.3	19.1	3226.9	48.5	78907.9
152	62393.1	1290.4	19341.3	1130.5	2566.4	595.1	119.0	12.8	3712.1	43.7	91204.3
153	57789.8	1114.5	8728.0	965.7	1901.9	387.4	327.0	10.3	2978.5	47.1	74249.9
154	50632.4	1005.0	21667.3	871.6	2423.0	576.8	440.8	16.8	3367.3	38.2	81039.1
155	49167.3	903.2	18125.0	964.6	1674.6	495.2	445.7	12.9	4283.9	44.6	76117.0
156	50592.8	1186.4	16879.9	988.3	2257.2	376.4	488.1	8.2	2750.6	44.8	75572.6
157	63071.6	1365.8	13036.0	1575.8	3380.7	581.6	1590.4	32.0	3456.8	51.7	88142.3
158	65880.3	1322.3	16325.2	1709.7	2050.1	625.4	352.4	16.6	4409.8	54.8	92746.4
159	54998.8	1021.3	15306.2	857.3	1719.3	427.9	152.5	11.8	3995.3	49.0	78539.4
160	53916.7	1179.4	11824.4	715.1	1672.0	504.8	343.5	18.6	3131.7	53.2	73359.2

161	70227.6	921.3	12154.1	1067.2	1353.9	454.4	246.7	11.2	4108.4	54.6	90599.5
162	52233.4	882.6	12987.4	1106.5	1358.6	435.2	259.2	9.3	3710.9	43.7	73026.7
163	70237.0	920.6	24865.9	1655.8	2739.2	628.1	364.7	12.0	4115.3	49.3	105587.9
164	66832.6	1210.7	23414.6	1565.8	2738.1	683.3	369.1	11.6	4056.8	49.2	100931.9
165	56443.9	940.7	16715.7	1988.3	1817.2	654.2	751.6	11.6	3713.7	45.1	83082.0
166	48178.6	657.4	15127.0	3156.3	1275.5	1013.7	163.9	12.3	3696.7	11.8	73293.1
167	65327.1	1236.0	19840.3	2027.3	2968.4	888.1	406.5	19.1	4024.7	44.5	96782.1
168	50141.3	1087.1	9999.6	1408.2	1087.3	499.8	411.8	13.5	3538.6	35.7	68223.0
169	52479.4	1659.1	19218.5	1050.6	2213.0	655.5	160.2	7.2	3489.1	48.9	80981.5
170	58959.0	1236.1	15396.2	1303.9	2238.6	581.4	226.3	7.5	3751.0	49.7	83749.8
171	53343.0	1407.6	17860.5	1196.9	1357.0	678.9	248.9	12.1	4785.2	49.0	80939.1
172	55368.6	1094.3	12786.9	1449.7	2120.1	492.5	317.3	9.0	3404.7	4.9	77047.9
173	62116.6	1450.5	17747.0	1294.6	1640.7	580.8	301.8	13.9	3965.4	53.9	89165.3
174	39681.0	967.1	12679.1	964.8	808.9	435.8	488.6	10.6	2172.1	42.6	58250.8
175	58905.3	997.0	9750.5	1159.7	1671.2	412.5	208.0	9.1	4608.9	46.5	77768.5
176	57347.5	1520.0	12122.5	2058.1	3418.9	683.1	1268.4	18.8	4160.3	53.1	82650.8
177	48160.3	1164.7	11784.3	1213.2	1308.5	443.2	552.0	9.4	3973.3	4.0	68612.9
178	47888.7	991.5	8941.1	1485.7	1945.5	390.6	921.5	21.7	4049.4	5.3	66641.0
179	67312.7	1481.6	8183.1	2925.3	3228.0	422.0	1416.9	11.2	4645.3	9.3	89635.5
180	63418.7	1274.0	12893.4	1619.3	1285.9	910.3	307.9	14.3	5783.1	9.8	87516.7
181	63923.8	940.9	20323.1	2007.7	1606.2	769.8	449.7	31.7	4540.2	7.0	94600.2
182	53251.7	964.0	12330.9	1122.8	1829.2	542.2	188.4	15.4	2900.1	62.4	73207.3
183	56935.5	1099.4	16936.7	907.6	2088.0	470.8	290.4	8.5	3957.7	57.8	82752.3
184	62636.6	1250.3	10373.0	1130.3	1644.8	479.8	613.7	11.3	2761.0	56.7	80957.4
185	52038.8	1348.0	18121.4	2081.5	2867.6	815.9	1494.7	10.3	4218.0	3.5	82999.7
186	39872.0	692.7	15974.3	759.2	1442.2	762.3	280.0	17.7	2765.5	33.9	62599.7
187	48290.4	944.8	14447.1	1258.9	1789.2	611.1	403.3	10.1	2818.2	4.9	70578.1
188	63460.7	1206.6	14244.5	683.7	2600.2	482.0	624.7	12.0	4246.3	59.0	87619.7
189	58932.8	1114.5	22769.6	1258.6	1553.5	790.2	255.1	16.5	4308.3	47.4	91046.6
190	64564.7	1259.8	15053.2	1097.1	2430.3	476.9	303.6	11.1	4441.7	59.0	89697.6
191	48197.4	1139.0	12452.1	957.1	1167.4	475.2	515.7	9.3	2626.1	51.8	67591.2

192	45610.4	809.4	12929.3	1025.5	1163.7	589.7	283.4	13.4	3647.9	50.4	66123.0
193	50896.3	1066.8	10274.6	640.4	3312.7	446.7	749.0	9.9	3236.0	48.1	70680.4
194	44513.2	607.4	16814.1	2834.6	1135.4	1176.2	280.1	23.5	2219.8	47.4	69651.8
195	84717.1	773.3	22369.5	3162.7	3315.9	1076.2	331.4	19.4	5105.7	70.1	120941.3
196	59304.9	1316.6	20300.1	2827.5	1974.1	895.6	379.9	21.3	4954.4	53.3	92027.7
197	58967.6	1030.6	9657.6	1667.7	2575.4	556.0	181.2	12.4	3726.5	47.6	78422.5
198	62924.0	1299.1	15472.8	1395.5	3948.1	583.2	224.5	11.3	4040.3	51.5	89950.2
199	64395.3	1326.8	14057.0	1319.1	3064.9	533.6	662.2	8.0	5212.5	56.7	90636.1
200	84170.7	874.6	20422.4	2296.9	3871.8	843.9	586.8	16.8	6248.2	69.0	119401.1
201	60235.9	1074.4	14554.0	973.8	1417.9	424.9	579.7	8.9	3320.4	52.3	82642.0
202	69387.8	1360.8	15327.4	971.0	1306.1	608.7	4614.4	92.6	4167.8	58.5	97895.1
203	52948.5	1003.7	10034.3	2499.8	2077.4	723.2	698.9	27.9	3159.5	38.3	73211.4
204	65587.2	1522.4	12685.9	1505.8	1950.2	695.2	669.9	15.1	3606.5	55.6	88293.9
205	72144.7	1303.7	15695.2	1830.7	4722.1	1042.3	275.8	27.7	4442.0	56.6	101540.8
206	70179.8	1697.3	14665.2	2021.8	2398.1	1015.8	857.9	85.3	3526.9	57.1	96505.1
207	56350.5	1248.3	11957.0	902.0	1771.9	703.6	454.1	19.2	3533.3	44.6	76984.5
208	86987.4	1713.2	17003.2	2472.3	1846.1	1097.4	125.7	34.4	5565.0	73.1	116917.8
209	70009.0	1381.5	12192.1	1746.9	2906.4	822.0	447.1	28.1	4037.4	58.8	93629.3
210	68310.8	1339.9	10573.0	1570.0	1602.0	601.7	151.7	21.5	4180.9	56.5	88408.0
211	87215.0	1525.4	13908.1	2858.0	3831.7	750.7	778.0	21.8	4671.1	76.2	115635.9
212	83002.6	1674.1	14152.5	1262.2	2197.6	711.8	847.5	30.8	4007.5	71.2	107957.7
213	77374.2	1544.7	15376.8	1556.7	2518.0	947.9	460.9	26.6	3802.7	58.4	103666.9
214	70722.9	2068.2	15139.8	2651.2	2373.7	1263.9	197.4	34.7	5801.4	56.6	100309.8
215	83454.1	1443.3	15090.5	3833.4	2248.0	721.9	1021.5	55.7	6592.2	74.2	114534.8
216	74648.9	1655.2	12062.6	2299.5	3137.2	901.9	438.0	33.5	4715.4	54.3	99946.6
217	71776.7	1500.8	10477.3	2371.8	2713.7	711.7	704.8	25.1	4644.4	66.1	94992.3
218	67521.7	1584.0	9891.2	2406.9	2135.6	557.7	86.5	18.9	3320.9	57.1	87580.5
219	109660.3	1702.2	15303.1	2727.6	3071.0	856.4	440.8	24.6	6530.4	95.9	140412.5
222	80178.4	1638.4	13485.1	2741.9	2924.7	921.0	500.8	22.8	4657.9	65.6	107136.5
223	65781.0	1218.4	8966.7	1224.4	1680.2	456.1	164.0	16.8	4128.1	50.0	83685.9
224	60900.0	1213.2	12514.5	1339.4	1756.0	621.9	200.0	17.1	3569.4	50.5	82181.8

229	73895.7	1550.6	13302.3	2016.0	4147.0	706.6	439.7	15.8	4963.2	54.6	101091.5
231	61141.5	988.4	11488.6	2789.3	1903.2	712.8	925.2	30.3	3783.4	59.1	83821.9
232	112096.5	1855.9	14564.3	1836.9	4636.0	559.0	685.9	9.8	7623.4	87.8	143955.4
233	73794.0	1089.5	14127.1	1814.7	2907.1	921.3	610.8	21.4	4551.7	54.5	99892.1
234	76221.3	1055.7	12991.3	2444.8	2031.9	770.8	418.6	18.1	4565.4	54.9	100572.8
235	67697.7	1933.7	8516.3	770.5	3676.2	380.4	430.0	19.5	3246.5	49.8	86720.7
236	62237.1	1061.9	10208.3	2315.7	1981.6	614.1	368.1	21.4	4230.1	48.2	83086.3
237	69699.2	1225.2	13816.0	2245.3	2242.5	824.0	635.4	22.6	4305.6	57.7	95073.6
238	85739.5	1594.8	14620.2	3088.0	4102.4	937.8	714.8	20.9	5410.3	80.2	116308.8
239	91365.9	1480.2	15939.0	3852.9	2516.0	973.6	575.6	11.8	6940.0	67.7	123722.6
240	76615.9	1594.1	13146.4	1977.9	2683.8	660.0	601.3	16.8	4576.4	64.9	101937.7
241	91839.9	1473.2	19020.7	1966.5	3433.2	1062.5	1023.0	23.6	5845.1	67.5	125755.1
242	82172.7	1246.6	15135.4	3293.1	3197.0	1060.2	670.0	22.2	5806.8	58.2	112662.1
243	67303.8	1279.5	12171.6	2148.0	2141.5	803.8	698.6	36.9	4087.9	58.5	90730.1
244	58715.9	1259.5	12796.7	1488.0	2724.9	756.7	490.3	19.5	3671.7	56.5	81979.7
245	68366.6	1389.6	10187.1	930.4	1384.4	485.4	3154.2	65.7	4189.5	60.4	90213.3
246	71594.5	1102.8	14410.4	2961.4	3717.6	1322.7	721.7	30.8	4422.3	64.2	100348.3
247	56115.6	1128.6	7575.5	1008.5	991.2	475.3	414.6	15.4	2872.6	53.1	70650.4
248	72221.2	1401.2	14080.2	2515.8	2123.4	942.5	487.9	20.9	5385.5	64.7	99243.1
249	71991.2	1354.0	12853.6	2866.2	3301.1	1247.9	2626.3	204.9	4006.7	70.4	100522.2
250	66481.2	1160.0	9759.6	2222.6	2219.8	818.4	388.8	19.0	3567.4	52.9	86689.8
251	89258.5	1628.0	13110.8	3357.8	2808.7	1030.3	554.5	31.0	4953.5	75.8	116808.8
252	77400.7	1535.6	11735.6	3169.6	2051.4	951.8	838.1	30.0	3771.2	58.3	101542.3
253	76551.6	2006.4	13612.8	1873.6	2461.2	1020.5	652.7	23.6	3802.8	62.6	102068.0
254	54581.1	813.9	9461.8	1042.3	1771.6	754.1	397.7	10.5	2458.4	46.4	71337.9
255	75269.2	1346.5	11746.8	1725.1	3739.6	768.0	602.9	16.9	4255.7	74.7	99545.4
256	75907.0	1093.5	14506.2	1563.2	1569.0	718.1	4364.7	150.2	3392.8	65.8	103330.4
257	54204.2	512.1	9126.0	1240.8	2073.0	465.0	616.6	11.2	2483.6	50.7	70783.2
258	71356.3	1452.6	13932.6	1068.5	2699.2	877.9	2785.4	146.6	3382.6	59.2	97760.9
259	81517.2	1316.0	12839.2	1821.7	2923.4	798.3	2279.5	110.0	3807.8	71.2	107484.2
260	74880.1	1618.9	12944.4	1469.3	4025.8	825.4	538.2	12.7	4219.9	55.3	100590.0

261	69144.6	1197.0	11253.4	1842.2	1990.0	756.7	412.1	35.0	4085.3	62.4	90778.7
262	85791.3	1232.7	9440.4	3217.5	2023.7	530.1	541.9	11.3	7306.3	67.7	110162.9
263	63452.0	955.5	10622.4	2306.7	1818.6	775.3	475.3	15.9	3854.4	56.8	84332.7
264	68564.6	1106.9	16274.3	1481.5	1778.0	1056.7	472.9	18.0	5098.6	56.3	95907.9
265	54907.2	904.4	9566.6	820.2	2197.3	525.5	296.2	21.3	3248.6	45.1	72532.3
266	73008.5	1097.8	8122.1	2465.5	2614.1	548.6	689.3	11.9	5078.3	53.6	93689.7
267	74771.3	1285.5	12939.6	2056.5	3046.0	1073.6	554.9	19.1	4845.4	54.2	100646.0
268	69663.2	1069.6	10573.8	2789.2	2395.5	724.1	1009.4	40.0	4713.7	64.3	93042.9
269	63734.9	1496.9	11271.1	2145.5	1791.9	823.8	390.1	19.5	4635.7	56.3	86365.7
270	73561.3	1275.3	12091.2	1537.0	1842.6	664.3	523.4	30.8	4098.1	63.0	95687.1
271	79547.4	1037.0	11934.6	1938.7	2571.0	833.7	718.3	19.5	3974.1	60.6	102634.9
272	58085.2	895.1	7931.4	1444.7	2028.0	536.5	310.8	20.3	2659.0	44.5	73955.6
273	76966.3	1402.2	10913.9	2357.1	2600.7	752.7	803.7	21.6	3478.3	65.2	99361.6
274	62223.5	1151.6	10784.2	1815.7	2800.5	693.8	578.7	22.1	2892.5	53.2	83015.8
275	85619.9	1180.3	14753.0	1983.6	2381.5	989.5	660.0	24.6	4289.2	57.5	111939.0
276	88431.4	1067.4	15560.1	3284.9	3123.8	1210.0	997.1	55.7	5542.9	68.3	119341.7
277	67322.3	980.4	9959.5	2051.6	1490.0	680.8	148.1	17.4	4419.0	60.5	87129.4
278	75998.2	920.1	12308.1	2531.7	2515.0	828.8	153.7	28.9	4995.3	68.9	100348.8
279	68284.6	1174.9	14892.0	2318.9	1962.0	1018.2	121.9	28.4	4206.3	66.8	94073.8
280	70793.5	1165.0	12092.3	1868.2	2721.0	588.7	127.3	17.1	3932.6	55.4	93361.1
281	90034.6	1502.7	16231.1	5530.4	2030.6	1366.2	401.0	32.2	5302.3	69.0	122500.3
282	80861.4	1290.2	15368.2	2900.1	2032.2	1143.5	502.8	43.3	4634.0	61.1	108836.8
283	74445.9	1418.7	15137.1	968.2	2838.2	986.6	126.6	26.1	4091.3	57.0	100095.9
284	58270.0	958.7	9619.2	1148.0	1868.6	438.4	309.9	35.0	2732.9	51.6	75432.2
285	69376.8	1020.3	11750.7	1629.2	3317.0	738.7	611.4	24.6	3511.7	59.3	92039.8
286	67100.4	1228.4	8059.4	3558.3	3141.6	542.4	131.2	30.3	3785.8	62.7	87640.6
287	78584.9	962.9	12657.9	1505.2	1609.4	702.8	371.4	20.7	4830.0	51.8	101297.0
288	62407.8	678.9	12109.7	1864.9	1634.8	896.9	1597.9	117.4	3280.0	53.5	84641.7
289	65880.3	643.0	19419.4	2315.2	1839.1	884.8	316.4	11.0	5138.3	58.4	96505.9
290	53754.9	1055.1	11250.3	1246.5	1987.9	413.0	524.0	10.7	3380.0	53.6	73676.1
291	44041.4	796.3	21204.8	615.0	2361.0	662.2	651.2	17.0	2139.5	37.9	72526.4

292	44515.8	746.7	20185.9	2061.8	2718.6	568.5	1527.8	11.7	4984.1	38.2	77359.0
293	77579.4	1268.0	19952.2	1628.8	3318.4	752.5	671.0	25.6	4598.7	57.0	109851.6
294	53117.0	835.8	13805.6	1508.4	1953.9	763.1	450.0	17.2	3977.2	42.7	76471.0
295	81882.7	1262.7	13460.3	1656.1	2278.7	503.4	171.7	9.9	5172.3	59.9	106457.6
296	47112.4	844.8	10523.3	829.6	2798.5	392.5	743.6	22.5	2416.8	45.1	65729.0
297	68257.5	998.3	11823.1	1441.0	2100.7	515.9	515.6	13.6	3127.7	65.2	88858.4
298	55569.8	782.8	20578.0	1569.8	1342.0	809.2	621.2	50.9	4054.7	39.5	85418.0
299	70926.1	1686.8	18516.9	1554.5	2364.6	741.4	253.8	13.5	4645.2	58.0	100760.6
300	38607.5	720.0	10566.4	538.0	2128.2	273.6	319.9	7.0	2221.1	37.8	55419.5
301	44358.4	891.0	14211.1	1046.7	1153.6	656.9	694.8	26.3	2519.8	40.6	65599.2

Supplementary Table S2.

Serum lipid level alterations in the Iberian GD and PD cohort using 30% cut-off on absolute lipid levels. A. Patient groups compared with the control group. Results obtained, when controlling for sex and age at blood collection. Patients' sex coded as 0 (female) or 1 (male). **B.** Association of PD disease severity, therapy and disease duration with lipid level changes. **C.** Association of GD therapy with lipid level changes. **A.-C.:** Results are presented as analyzed by method #1 (see "Quantification of the levels of lipid groups and their species"). Lipid nomenclature as in the text. Effects of the independent variables on the normalized lipid levels (dependent variables) are expressed by the corresponding estimated regression coefficients, with positive (negative, respectively) coefficients meaning that higher values of the independent variables are associated with higher (lower, respectively) values of the lipid levels. All variables were scaled so that their maximum absolute value was equal to 1. *, ** and *** denote P < 0.05, P < 0.01, and P < 0.001, respectively.

**Phospholipids**	**PC**	**LPC**	**PC/LPC**	**P-PC**	**PE**	**LPE**	**PE/LPE**	**P-PE**	**PS**	**LPS**	**PS/LPS**	**PI**	**PG**
**Between-group analyses**													
**A. Patient groups vs. control group**													
**Intercept**	0.531***	0.560***	0.977***	0.431***	0.149**	0.363***	0.467***	0.391***	0.096**	0.099**	1.015***	0.464***	0.288***
**PD (n=100)**	**0.043****	**-0.115*****	**0.280*****	-0.037	0.033	**0.052***	0.006	**0.088*****	**-0.032****	0.005	**-0.480*****	0.008	0.025

**GBA-PD (n=15)**	0.024	**-0.104*** **	**0.235*** **	-0.027	0.026	0.012	0.031	0.055	-0.015	0.003	-0.304	-0.007	0.026
**GD (n=70)**	**0.076*** **	**-0.064*** **	**0.250*** **	-0.021	**0.070** **	**0.088*** **	0.029	**0.063***	-0.004	**0.056** **	-0.203	0.0268	**0.0697*** **
**Sex**	0.011	0.001	0.006	0.020	0.029	0.018	0.039	0.029	0.016	0.0189	0.086	-0.009	0.013
**Age of Collection**	0.017	0.0358	0.024	0.115	**0.169** **	0.081	**0.251***	0.062	-0.014	-0.010	0.0364	0.087	7.20e-05
**Within-group analyses**													
**B. Associations with PD disease severity, medication and disease duration (*n* = 46; PD patients)**													
**Intercept**	0.786***	0.669***	1.167***	0.363*	0.383*	0.656***	0.570	0.760***	0.0599	0.0887	0.966*	1.037***	0.565**
**Hoehn and Yahr scale**	-0.039	-0.052	0.043	-0.149	0.056	-0.0008	0.127	-0.163	0.008	-0.042	0.449	0.036	-0.035
**Dopamine Agonist**	**-0.118***	-0.091	0.002	0.017	-0.124	-0.102	-0.152	0.075	-0.040	0.028	-0.485	**-0.204***	-0.081
**Anti-Parkinsonian**	-0.024	-0.009	-0.022	-0.088	-0.071	0.0142	-0.172	0.0003	0.015	**0.060*** **	-0.075	-0.077	0.0987
**Sex**	-0.038	-0.0005	-0.106	0.023	-0.038	-0.025	-0.068	-0.027	0.021	0.002	0.178	**-0.106** **	-0.014
**Age of Collection**	-0.124	-0.132	0.064	0.177	0.084	-0.088	0.287	-0.269	0.006	-0.001	-0.410	**-0.376***	-0.222
**Disease Duration**	-0.070	**-0.142***	0.304	-0.141	-0.030	-0.146	0.298	-0.167	0.0008	-0.017	0.211	-0.113	-0.094
**C. Associations with GD therapy [(n = 42) vs non-treated (n=27) GD patients]**													
**Intercept**	0.510***	0.383**	1.379***	0.430**	-0.077	0.233**	0.187	0.358*	-0.049	0.196	-0.867	0.353***	0.319***
**Imiglucerase (n=12)**	0.016	-0.053	0.099	0.026	-0.038	0.013	-0.067	-0.031	-0.014	-0.009	-0.243	-0.017	0.016

**Velaglucerase alfa (n=16)**	0.062	0.008	0.038	0.081	**0.093***	**0.135****	-0.036	0.014	-0.005	0.031	-0.137	0.031	0.028
**Eliglustat (n=6)**	0.091	-0.010	0.105	0.115	0.051	0.034	0.032	0.125	-0.018	-0.074	0.058	0.100	**0.093*****
**Miglustat (n=8)**	-0.035	0.084	**-0.250***	-0.029	-0.074	0.012	-0.184	-0.035	-0.032	0.030	-0.174	-0.021	-0.025
**Sex**	0.039	**0.069***	-0.071	-0.010	0.101	0.068	0.095	**0.076***	0.013	0.042	0.360	0.060	0.015
**Age of Collection**	0.104	0.147	-0.144	0.067	**0.506***	0.290	0.676	0.157	0.183	-0.080	2.201	0.219	0.031

Supplementary Table S3.

Serum lipid level alterations in the Iberian GD and PD cohort using relative lipid levels. A. Patient groups compared with the control group. Results obtained, when controlling for sex and age at blood collection. Patients' sex coded as 0 (female) or 1 (male). **B.** Association of PD disease severity, therapy and disease duration with lipid level changes. **C.** Association of GD therapy with lipid level changes. **A.-C.:** Results are presented as analyzed by method #1 (see "Quantification of the levels of lipid groups and their species"). Lipid nomenclature as in the text. Effects of the independent variables on the normalized lipid levels (dependent variables) are expressed by the corresponding estimated regression coefficients, with positive (negative, respectively) coefficients meaning that higher values of the independent variables are associated with higher (lower, respectively) values of the lipid levels. All variables were scaled so that their maximum absolute value was equal to 1. *, ** and *** denote $P < 0.05$, $P < 0.01$, and $P < 0.001$, respectively.

**Phospholipids**	**PC**	**LPC**	**PC/LPC**	**P-PC**	**PE**	**LPE**	**PE/LPE**	**P-PE**	**PS**	**LPS**	**PS/LPS**	**PI**	**PG**
**Between-group analyses**													
**A. Patient groups vs. control group**													
**Intercept**	0.723***	0.172***	4.408***	0.012***	0.011***	0.007***	0.467***	0.022***	0.008**	0.0002***	0.611***	0.043***	0.0005***
**PD (n=100)**	**0.035****	**-0.038*****	**1.268*****	**-0.002*****	0.002	**0.0007***	0.009	**0.004*****	-0.002	1.54e-05	**-0.209*****	-0.0006	2.5e-05

**GBA-PD (n=15)**	0.031**	-0.032***	1.085***	-0.0008	0.001	7.23e-05	0.032	0.003	-0.002	1.12e-05	-0.166	-0.0007	4.13e-05
**GD (n=70)**	0.030***	-0.031***	1.131***	-0.002***	0.002*	0.0007*	0.030	0.001	0.0006	0.0001**	-0.092	-0.002	7.996e-05**
**Sex**	-0.0001	-0.002	0.019	0.0002	0.001	0.0001	0.039	0.001	0.001	3.5e-05	0.030	-0.002	9.99e-06
**Age of Collection**	-0.016	-0.001	0.128	0.003*	0.008*	0.0004	0.250*	0.002	-0.002	-4.7e-05	0.016	0.0051	-1.78e-05
**Within-group analyses**													
**B. Associations with PD disease severity, medication and disease duration (*n* = 46; PD patients)**													
**Intercept**	0.693***	0.167***	4.032***	0.0009	0.014	0.010**	0.358	0.028*	0.014	0.0003	1.196*	0.071***	0.0007**
**Hoehn and Yahr scale**	0.003	-0.006	0.212	0.004	-0.001	-0.0005	-0.047	0.009	-0.003	5.52e-05	-0.311	-0.006	-0.0001
**Dopamine Agonist**	0.012	-0.017*	0.870*	0.003*	0.0002	-0.0009	0.077	0.004	0.002	7.78e-07	0.073	-0.003	0.0001
**Anti-Parkinsonian**	0.007	-0.008	0.437	0.0009	-0.0007	-0.0007	0.027	4.64e-05	0.002	-5.6e-05	0.186	5.83e-05	0.0001
**Sex**	-0.001*	0.007	-0.373	0.002	-0.002	-0.0002	-0.057	0.0004	0.0002	-3.97e-06	0.013	-0.006*	3.95e-05
**Age of Collection**	0.036	-0.015	0.679	0.009	0.01	-0.0003	0.364	-0.009	-0.011	-2.66e-05	-0.874	-0.020	-0.0002
**Disease Duration**	0.025	-0.014	0.830	-0.003	0.002	-0.0008	0.258	-0.007	-0.001	5.36e-05	-0.153	-0.001	-0.0002
**C. Associations with GD therapy [(n = 42) vs non-treated (n=27) GD patients]**													
**Intercept**	1.001***	0.427**	6.221***	0.598***	0.017	0.465**	0.165	0.513**	0.012	0.267	-0.520	0.541***	0.926***
**Imiglucerase (n=12)**	0.026	-0.053	0.418	0.028	-0.044	0.034	-0.0688	-0.064	0.020	-0.012	0.012	-0.030	0.031

**Velaglucerase alfa (n=16)**	0.012	-0.041	0.165	0.040	0.065	**0.106***	-0.039	-0.054	0.015	0.017	-0.030	-0.022	-0.003
**Eliglustat (n=6)**	0.018	-0.058	0.443	0.043	0.014	0.002	0.018	0.038	-0.046	-0.088	0.431	0.018	0.075
**Miglustat (n=8)**	-0.024	0.089	**-1.133***	-0.025	-0.090	0.029	-0.172	-0.023	-0.036	0.041	-0.406	-0.007	-0.035
**Sex**	-0.020	0.026	-0.323	**-0.060***	**0.085***	0.013	0.095	0.063	0.029	0.031	0.307	0.026	-0.033
**Age of Collection**	-0.089	0.069	-0.611	-0.033	**0.535***	0.147	0.631	0.128	0.210	-0.149	2.266	0.161	-0.124
